# Interpretations of Studies on SARS-CoV-2 Vaccination and Post-acute COVID-19 Sequelae

**DOI:** 10.1097/EDE.0000000000001720

**Published:** 2024-04-18

**Authors:** Bronner P. Gonçalves, Piero L. Olliaro, Peter Horby, Laura Merson, Benjamin J. Cowling

**Affiliations:** From the a ISARIC, Pandemic Sciences Institute, University of Oxford, Oxford, United Kingdom; b Department of Comparative Biomedical Sciences, School of Veterinary Medicine, University of Surrey, Guildford, United Kingdom; c WHO Collaborating Centre for Infectious Disease Epidemiology and Control, School of Public Health, Li Ka Shing Faculty of Medicine, The University of Hong Kong, Hong Kong Special Administrative Region, People’s Republic of China.

**Keywords:** Causal inference, Long COVID, Principal stratification, Vaccination

## Abstract

This article discusses causal interpretations of epidemiologic studies of the effects of vaccination on sequelae after acute severe acute respiratory syndrome coronavirus 2 infection. To date, researchers have tried to answer several different research questions on this topic. While some studies assessed the impact of postinfection vaccination on the presence of or recovery from post-acute coronavirus disease 2019 syndrome, others quantified the association between preinfection vaccination and postacute sequelae conditional on becoming infected. However, the latter analysis does not have a causal interpretation, except under the principal stratification framework—that is, this comparison can only be interpreted as causal for a nondiscernible stratum of the population. As the epidemiology of coronavirus disease 2019 is now nearly entirely dominated by reinfections, including in vaccinated individuals, and possibly caused by different Omicron subvariants, it has become even more important to design studies on the effects of vaccination on postacute sequelae that address precise causal questions and quantify effects corresponding to implementable interventions.

Vaccines that target severe acute respiratory syndrome coronavirus 2 (SARS-CoV-2) have been consistently shown to protect against acute manifestations of coronavirus disease 2019 (COVID-19).^1–6^ Quantifying their effects on the long-term sequelae that develop following SARS-CoV-2 infection—their effects on the risk of Long COVID or postacute COVID-19 syndrome (PACS)—is, however, more complex. Informative answers to the question of whether vaccination reduces the risk or severity of PACS require recognizing that there are at least four different possible interpretations for it^7,8^: (1) Does SARS-CoV-2 vaccination reduce the risk of PACS related to a potential future infection in a population of individuals with no history of SARS-CoV-2 infection? (This question relates to the total effect of vaccines on PACS, including via paths mediated by infection.) (2) Does SARS-CoV-2 vaccination of individuals with no history of infection at the time of vaccine administration reduce the risk of PACS conditional on acquiring the infection that might cause PACS in those patients? (3) Does vaccination after SARS-CoV-2 infection reduce the prospective risk of PACS linked to the same infection? (4) In patients with PACS diagnosis, can vaccination accelerate recovery? In Figure 1, we present, for each interpretation, the temporal ordering of vaccination, infection, and PACS. We argue that interpretations (1), (3), and (4) of the question can be answered by analyses with causal estimands; interpretation (2), however, cannot be understood as causal.

**FIGURE 1.**
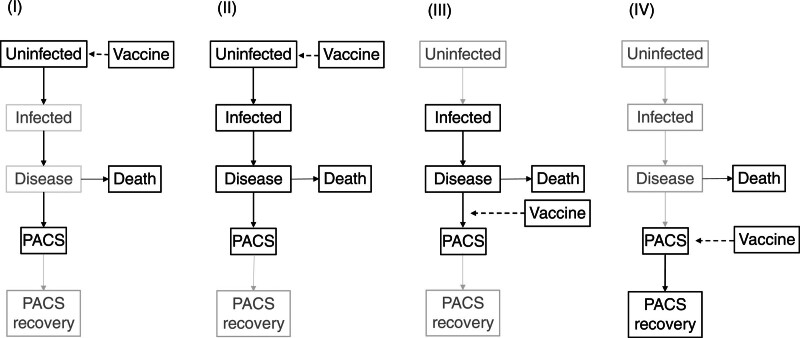
Timing of exposure (vaccination) in relation to infection and postacute COVID syndrome (PACS) development. In Panels I–IV, we present, for the four interpretations of our research question, both a simplified sequence of pathologic states, and the timing of vaccination. Information on states colored in gray is not used in analyses of each corresponding research question. Note that these analyses condition on patients not dying, and this is represented by the “Death” boxes. Although, in this figure, interpretation (1) refers to individuals with no previous exposure to SARS-CoV-2 at the time of vaccination, it is equally relevant for patients with previous infections and who are at risk of PACS caused by reinfection.

We start by discussing interpretation (1), which corresponds to the total effect of vaccination on PACS. Figure 2A shows a directed acyclic graph, where we present assumptions on the causal structure of the relation between vaccination and PACS. Vaccination has been shown, both in trials and observational studies, to be associated with a reduced risk of several SARS-CoV-2 outcomes, including infection and clinical disease, which are necessary or possible steps in the development of PACS. Although confounding is a common problem in observational studies,^9^ as some factors are upstream in the causal pathway of both exposure (vaccination) and outcome (PACS), appropriately accounting for a sufficient set of confounders would allow unbiased effect estimation. Causal diagrams relevant to interpretations (3) and (4) of the question are different (Figure 2B): the effect of vaccination that is the target of the estimation in these studies does not involve infection prevention; rather, the effect of interest relates to the reduction of sequelae in individuals who developed infection and survived acute disease. Note that in this case, although analyses involve conditioning on SARS-CoV-2 infection or COVID-19, the relevant exposure is postinfection vaccination.

**FIGURE 2.**
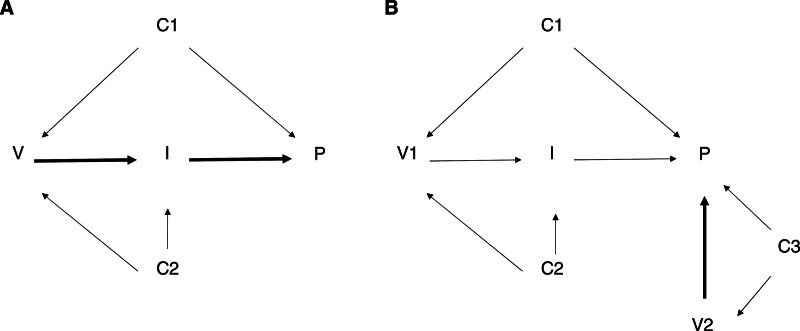
Causal diagrams relevant to the formulations (1) (A) and (3) (B) of the question on SARS-CoV-2 vaccination and postacute COVID syndrome (PACS). A, the vaccine effect on PACS is, for simplicity, assumed to be entirely mediated by the effect on infection; confounders of the association between vaccination (V) and infection (I), confounder C2, and of the association between V and PACS (P), confounder C1, are also shown. B, vaccination before infection is represented by V1, and vaccination after infection, by V2; the causal effect of interest here is V2 → P. Note that confounder C3 might bias the association between V2 and P. In both panels, thicker arrows indicate the directed path of interest.

To discuss whether formulation (2) of our question—that is, whether preinfection vaccination modifies PACS risk conditional on resolution of SARS-CoV-2 infection—can be considered a valid causal question, we first need to consider a view on causal effects that is prevalent in the epidemiology research community. According to this view,^10–13^ a valid, interpretable quantitative notion of causal effects is one that corresponds to, or is expressible as, well-defined hypothetical interventions. Estimands in studies that use interpretation (1), (3), or (4) conform to this definition and are thus causal. For studies using interpretation (2) it is difficult to conceive an intervention that would change preinfection vaccination status without affecting infection status; in this case, the effect is not well defined. Another argument against a causal interpretation of formulation (2) relates to the fact that the individuals who would develop infection or disease are not the same for the different exposure levels. Or put in terms of counterfactuals, under the consistency condition, the estimand, in this case, compares PACS risk for those who would have developed infection if vaccinated to those who would become infected if they did not receive vaccination or who would have become infected even if vaccinated; there is not a one-to-one correspondence between individuals in these two sets. In fact, this comparison can be viewed as related to conditioning on a post-treatment variable and only corresponds to a causal estimand under the principal stratification framework. As described by Frangakis and Rubin,^14^ and by Hudgens and Halloran^15^ in the context of vaccination, analyses that condition on post-treatment variables can be interpreted in causal terms within strata defined by the joint potential outcomes of the post-treatment variable under different exposure levels (for interpretation [2], the post-treatment variable corresponds to SARS-CoV-2 infection or disease). The estimand that compares PACS frequencies in vaccinated and unvaccinated groups would only have a causal interpretation in the stratum of the population that consists of individuals who would acquire SARS-CoV-2 infection, and potentially develop clinical disease, regardless of vaccination status. Using potential outcomes notation, these studies would only have a causal interpretation for individuals with the following joint potential infection outcomes:


$$\begin{array}{l} \left(I_{j}^{v=1}=1,I_{j}^{v=0}=1\right), \end{array}$$

where $I_{j}^{v}$ corresponds to the potential outcome of individual *j* for the post-treatment variable (infection) under exposure level (preinfection vaccination status) *v*. Although this is the only principal stratum in which potential PACS outcomes are defined for both exposure levels, assuming a binary post-treatment variable, there are three other principal strata, corresponding to different joint potential infection outcomes. For a multi-sided discussion on the value of the principal stratification framework in different contexts, for example, noncompliance, see the following articles.^16–18^ Note that although this is an appropriate framework to discuss studies where investigators do not control the post-treatment variable, for studies in which it is possible to manipulate the post-treatment variable, for example, infection challenge studies,^19^ other causal questions might be more relevant.^20,21^

Care must then be exercised when interpreting studies on PACS and vaccination. If the target population of the analysis is the population of all individuals eligible for vaccination, the nonconceivability of a hypothetical intervention under interpretation (2) implies that these studies on preinfection vaccination and PACS that condition on infection do not have a causal interpretation. For these analyses, if the objective is to estimate causal vaccine effects in the stratum of individuals who would be infected with SARS-CoV-2 regardless of vaccination, then the effects would only apply to an unidentifiable (only one of the two potential infection outcomes can be observed for an individual) subset of the population for whom vaccines would not prevent infection. Estimation and identification conditions of this principal causal effect are discussed in reference 15. In the Table, we illustrate causal vaccine effects in the doomed principal stratum and show that for some combinations of parameters, a higher frequency of PACS in the vaccinated compared to the unvaccinated group could be consistent with a protective effect in that stratum.

**TABLE.** Quantitative examples of causal vaccine effects on post-acute COVID syndrome (PACS) conditional on SARS-CoV-2 infection in the group of individuals who would be infected regardless of vaccination status (interpretation [2]; in the table, the causal effect is represented by $VE_{PACS-d}$)

	Frequency of PACS (%)				
Study	Vaccinated Group	Unvaccinated Group	β	$VE_{inf}$	$VE_{PACS-d}$
A	30	41.8	1.1	30	29.4
A	30	41.8	1.1	70	30.9
A	30	41.8	1.5	30	32.9
A	30	41.8	1.5	70	38.5
B	13.1	11.6	1.1	30	<0
B	13.1	11.6	1.1	70	<0
B	13.1	11.6	1.5	30	<0
B	13.1	11.6	1.5	70	10.7

In this table, we present the results of calculations of possible vaccine effects in the doomed principal stratum. We used frequencies of PACS reported in two previous studies (in the table, studies A^22^ and B^23^); as these calculations are only illustrative, in choosing these values, we ignored some aspects of these studies (e.g., the number of vaccine doses being compared, study-specific definitions of PACS, or whether studies excluded patients hospitalized during acute infection). For one of these studies, frequencies of PACS by vaccination status were not reported and were calculated from one of the tables in the manuscript. Assumptions on the effect of vaccination on the post-treatment variable were necessary; thus, for each study, we assumed two different values for vaccine efficacy against infection ($VE_{inf}$). We also assumed different values for a parameter that corresponds to the odds ratio (β) of PACS under no vaccination in the doomed principal stratum versus the stratum of the population for which vaccination is protective. Discussion on the methodology is included in the eAppendix; http://links.lww.com/EDE/C112.

A recent systematic review^8^ included primarily studies on the association between preinfection vaccination and PACS conditional on infection, and studies on PACS recovery after postinfection vaccination. In another systematic review on vaccination and PACS,^7^ most studies included were consistent with the interpretation (2) of our original question and reported an association between lower PACS risk and preinfection vaccination in individuals with a history of infection. For example, analyses using a large database from the US Department of Veterans Affairs estimated a lower risk of PACS symptoms in patients who had been vaccinated before SARS-CoV-2 infection compared to those who did not receive preinfection vaccination (hazard ratio 0.85; 95% confidence interval = 0.82, 0.89). Studies designed to answer the research questions under interpretations (3) and (4) were also included in the latter review and support the hypothesis that postinfection vaccination can prevent and accelerate recovery from PACS. For example, in the United Kingdom, a vaccine dose after SARS-CoV-2 infection was associated with an initial decrease in the likelihood of PACS symptoms.^24^ Consistent with this, the results of a French study^25^ of patients reporting PACS indicate that the receipt of a first vaccine dose was associated with a higher rate of remission of symptoms compared to patients who did not receive vaccination after PACS diagnosis.

One of the tasks of epidemiologists is to suggest theory-backed, sensible explanations for patterns in observational data so that analyses can be used in an appropriate manner by policy makers. Our aim here relates to that task—to discuss possible interpretations of observational studies on PACS and SARS-CoV-2 vaccines. In particular, we argue that most studies included in recent systematic reviews on this association correspond to the interpretation (2) described above and cannot be interpreted causally. Earlier during the pandemic, quantification of the total protective effect of pre-infection vaccination on PACS risk (interpretation [1]) would have provided additional evidence to support vaccine use. In the current epidemiologic situation, where individuals have often been repeatedly vaccinated and/or infected, a comparison relevant for public health planning might be one that estimates the effect of additional vaccine doses on the risk of PACS due to recent and potential subsequent reinfections—that is, an estimand that would combine ideas from interpretation (1), with regard to reinfections, and from interpretations (3) and/or (4) (see eAppendix; http://links.lww.com/EDE/C112 for additional discussion). Note also that SARS-CoV-2 vaccination itself, without infection, has been linked to PACS-like illness,^26^ which, if confirmed by future epidemiologic studies, would need to be taken into account when interpreting the different analyses discussed here.

## Supplementary Material


