# Supplementary material for: Interpretations of Studies on SARS-CoV-2 Vaccination and Post-acute COVID-19 Sequelae
Source: Epidemiology. 2024 Apr 18;35(3):368–71. doi: 10.1097/EDE.0000000000001720 (PMC11191047; doi:10.1097/EDE.0000000000001720)
Supplement: Supplementary file 1 [file ede-35-368-s001.pdf]

Supplementary Appendix

Manuscript: Interpretations of studies on SARS-CoV-2 vaccination and post-acute COVID-19 sequelae

Discussion on the time 0 of follow-up

The different types of study described in the main text that have a causal interpretation (namely, the types I, III, IV) have different eligibility criteria. The less restrictive is the one that quantifies the total effect of vaccination on PACS, including via paths that include infection (type I); the other types have infection or COVID-19 (type III), or PACS (type IV) in their eligibility criteria. To avoid immortal time bias [1], the time 0 of the follow-up should be aligned with the time of exposure assignment and with the time when eligibility criteria are met. For studies that correspond to interpretation I, if these studies are to be restricted to patients with no history of infection before vaccination, although the time 0 of the follow-up should be aligned with the time of vaccination, or be set to a few weeks after vaccination to account for delay in the development of vaccine-induced immunological responses, and an equivalent time in the unexposed group, pre-vaccination data on infection history would allow to assess eligibility. For studies of the type III, the exposure of interest is post-infection vaccination, and for this reason, the time 0 of the follow-up should be aligned with the time of post-infection vaccination, and a matched or equivalent time in the unexposed group. In study type IV, the exposure of interest is vaccination after PACS diagnosis; the appropriate time 0 of the follow-up would, in this type of study, also be aligned with vaccination time.

Future studies on SARS-CoV-2 vaccination, reinfections and PACS

A question that is likely to become most relevant for public health policy relates to the total protective effect of additional vaccine doses against long-term sequelae in populations where reinfection is a common event. We can conceive at least two types of studies that could answer this causal question during future infection surges.

1) If, for example, the objective is to use observational data to assess the potential impact of vaccination early during future SARS-CoV-2 waves, one possibly useful type of study would compare, ideally stratifying by COVID-19 and vaccination history, individuals who receive vaccine doses at the start of a new SARS-CoV-2 infection wave with those with no recent vaccination; the interpretation of these studies is analogous to the interpretation I in the main text, except that it potentially (and in some settings, primarily) involves inclusion of previously exposed individuals.

2) If the objective of investigators is rather to assess the benefit of additional vaccine doses in individuals who have recently been (re)infected, e.g. during a prolonged SARS-CoV-2 wave, a study that compares individuals by post-recent infection vaccination status would be informative. In this second type of study, the protective effect that would be quantified would presumably be mediated via paths that involve prevention of future infections (and PACS associated with these future infections) as well as paths that are relevant for interpretations III and IV (that is, paths related to PACS that develops as a consequence of the [re]infection required for study eligibility).

Vaccine effects on PACS in the doomed principal stratum

Here, we present equations used to generate Table 1. These equations were described in the paper by Hudgens and Halloran [2], and below, we use similar notation. The first of these equations corresponds to the definition of vaccine efficacy against PACS in the doomed principal stratum (VE_{PACS-d}):

$$VE_{PACS-d} = 1 - \frac{\phi_{1.}}{\phi_{.1}} \qquad \text{(I)}$$

where $\phi_{1.}$ corresponds to $P[Y^{v=1} = 1 \mid I^{v=1} = 1,\ I^{v=0} = 1\,]$, and $\phi_{.1}$, to $P[Y^{v=0} = 1 \mid I^{v=1} = 1,\ I^{v=0} = 1\,]$. I^{v} and Y^{v} correspond to the potential infection and PACS outcomes, respectively, under vaccine assignment *v.*

The second equation used in the calculations defines the frequency of PACS in the unvaccinated group as a function of the frequencies of this outcome, under no vaccination, in two principal strata –the doomed principal stratum, and the stratum for which vaccination is protective – and of the vaccine efficacy against infection:

$$\tau_u = \gamma_1 VE_{inf} + \phi_{.1}(1 - VE_{inf}) \qquad \text{(II)}$$

where τ_u corresponds to the frequency of PACS in unvaccinated patients conditional on the potential infection outcome under no vaccination being infection; γ_1 corresponds to the probability of PACS in the stratum $\{j: I_j^{v=1} = 0, I_j^{v=0} = 1$ under no vaccination; and VE_{inf} represents vaccine efficacy against infection. The equation reflects the fact that individuals with recent infection and who were not vaccinated before infection are either part of the doomed or preventive (as defined in the previous sentence) principal strata.

Finally, we used the odds ratio of developing PACS, under no vaccination, in the doomed principal stratum versus the stratum for which vaccination is protective:

$$\beta = \frac{\frac{P[Y^{v=0}=1 \mid I^{v=1}=1, I^{v=0}=1]}{(1- P[Y^{v=0}=1 \mid I^{v=1}=1, I^{v=0}=1])}}{\frac{P[Y^{v=0}=1 \mid I^{v=1}=0, I^{v=0}=1]}{(1- P[Y^{v=0}=1 \mid I^{v=1}=0, I^{v=0}=1])}} = \frac{\phi_{.1}/(1-\phi_{.1})}{\gamma_1 / (1- \gamma_1)} \qquad \text{(III)}$$

Assuming VE_{inf} and β are known, we can calculate $\phi_{.1}$ and VE_{PACS-d} from a quadratic equation obtained by substituting $\phi_{.1}$ (from equation III) in equation II.

References

1. Hernan, M.A., et al., *Specifying a target trial prevents immortal time bias and other self-inflicted injuries in observational analyses.* J Clin Epidemiol, 2016. **79**: p. 70-75.
2. Hudgens, M.G. and M.E. Halloran, *Causal Vaccine Effects on Binary Postinfection Outcomes.* J Am Stat Assoc, 2006. **101**(473): p. 51-64.
